# Transient left bundle branch block and poor atrioventricular conduction during ablation of accessory pathway at the left ventricle

**DOI:** 10.1002/joa3.12440

**Published:** 2020-10-23

**Authors:** Kuo‐Feng Chiang, Chi‐Yen Wang, Jin‐Long Huang, Yu‐Cheng Hsieh

**Affiliations:** ^1^ Cardiology Division Asian University Hospital Taichung Taiwan; ^2^ Cardiovascular Center Taichung Veterans General Hospital Taichung Taiwan; ^3^ Institute of Clinical Medicine, and Cardiovascular Research Institute Department of Medicine School of Medicine National Yang‐Ming University Taipei Taiwan

**Keywords:** atrioventricular node, catheter ablation, left bundle branch block, Trans‐aortic approach, Wolff‐Parkinson‐White syndrome

## Abstract

A 56‐year‐old female with manifest Wolff‐Parkinson‐White (WPW) syndrome was sent to emergency room because of preexcited atrial fibrillation (AF) and became sinus rhythm after cardioversion. Then, she received catheter ablation of a left‐sided lateral accessory pathway. The patient immediately developed Wenckebach atrioventricular (AV) block and left bundle branch block (LBBB) during the initial ablation. The ECG still showed LBBB 1 hour after ablation. The LBBB became narrow QRS (The QRS complex in the electrocardiogram. The QRS complex includes the Q wave, R wave, and S wave) 1 day later. Two weeks later, Holter's ECG showed normal sinus rhythm with 1:1 AV conduction even at the maximum heart rate of 125 beats/min. Transient LBBB and poor AV nodal conduction could occur during ablation by the trans‐aortic approach.

## Graphical abstract

A 56‐year‐old female with manifest Wolff‐Parkinson‐White (WPW) syndrome was sent to emergency room because of atrial fibrillation with rapid wide QRS tachycardia and became sinus rhythm after cardioversion. After she received catheter ablation of a left‐sided lateral accessory pathway for 1 hour, the ECG showed LBBB, and the LBBB became narrow QRS 1 day later. Eliminating the accessory pathway could recover the AV nodal and His‐Purkinje conduction.

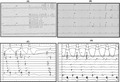

## 1 INTRODUCTION

Poor atrioventricular (AV) conduction and left bundle branch block (BBB) are rare complications of catheter ablation procedures involving areas distal to the AV node. We present a rare case of transient poor AV node conduction and LBBB, which occurred during ablation of a left‐sided posterolateral (LPL) accessory pathway by trans‐aortic approach in a patient with manifest Wolff‐Parkinson‐White (WPW) syndrome.

## 2 CASE REPORT

**FIGURE 1:**
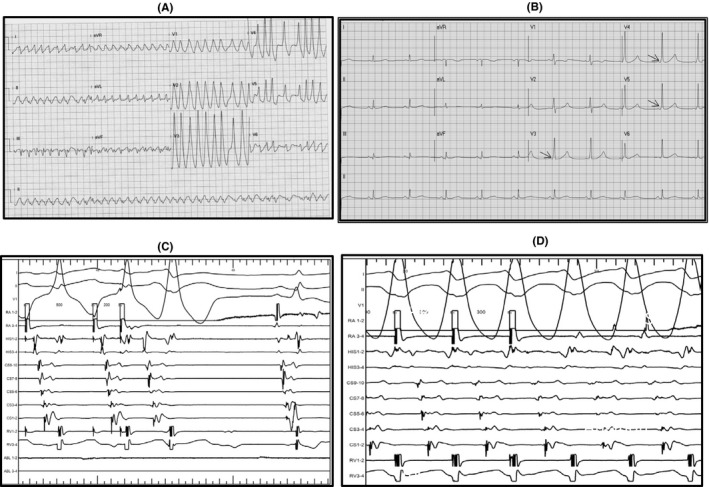
A, Irregular wide QRS tachycardia; B, Minimal delta wave (arrow) was found after cardioversion. C, Baseline EP study showed nearly full preexcited delta wave by the atrial pacing. D, HRA burst pacing (300 msec) induced nonsustained wide QRS tachycardia

**FIGURE 2:**
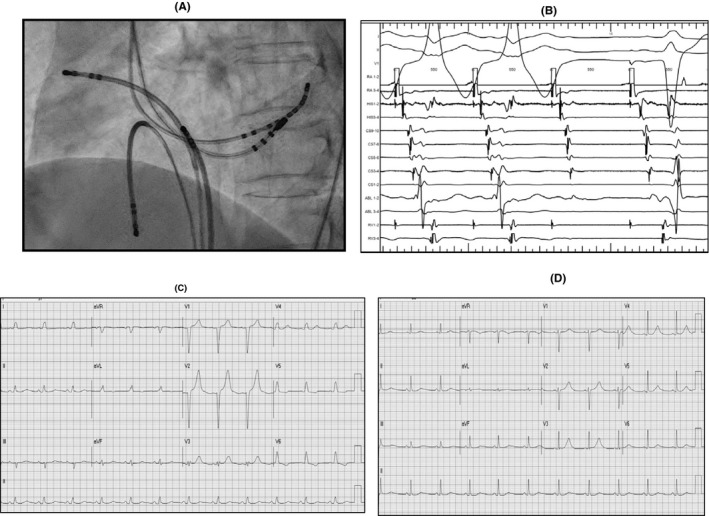
A, Ablation site at the left posterior‐lateral wall of left ventricle. B, Initiation of ablation showed the loss of delta wave and left bundle branch block (LBBB). C, One hour later, ECG still showed LBBB. D, One day later, ECG showed sinus rhythm with narrow QRS duration

A 56‐year‐old female was sent to emergency room with sudden collapse caused by polymorphic wide QRS (The QRS complex in the electrocardiogram. The QRS complex includes the Q wave, R wave, and S wave) tachycardia (Figure 1A), which was diagnosed as atrial fibrillation (AF) in WPW syndrome. Patients of preexcited AF with rapid ventricular response and unstable hemodynamics should receive prompt direct‐current cardioversion. After the cardioversion, the ECG showed minimal delta wave in the precordial leads (Figure 1B). She was referred to our cardiovascular center for electrophysiological (EP) study and radiofrequency ablation (RFA). Baseline cycle length: 814 ms, AH interval: 86 ms, HV interval: 32 ms, antegrade accessory pathway (AP) 1:1: <300 ms, AP effective refractory period (ERP): <200/500 ms, and atrial effective refractory period <200/500 ms Retrograde AP 1:1 = 350 ms and ERP: 340/500 ms Initial EP study showed nearly full preexcited delta wave by atrial pacing 500 msec in Figure 1C. Baseline EP study was difficult to find the AV nodal conduction under this dominant antegrade accessory pathway. Short run of PAT with fully preexcited delta wave tachycardia could be induced (Figure 1D). After initiation of RFA at the LPL wall of left ventricle (LV) (Figure 2A), transient Wenckebach AV block and LBBB were found under atrial continuous pacing of 550 msec (Figure 2B). Five minutes later, ECG still showed LBBB and we rechecked the characteristics of AV node conduction which showed AVN 1:1 = 580 ms and AVN ERP: 470/600 ms Then, continuous RFA was performed at the LPL of LV to eliminate the accessory pathway completely. However, ECG still showed LBBB with normal PR interval 1 hour after ablation (Figure 2C). The LBBB became narrow QRS after 1 day (Figure 2D). The Holter's ECG showed sinus rhythm with mean heart rate (HR): 60/min (44‐125 beats/min), with neither BBB nor AV block.

## 3 DISCUSSION

This case was instructive for two reasons. First, LBBB and impairment of AV node conduction could occur when the ablation catheter was inserted into the LV by the trans‐aortic approach. Secondly, poor AV nodal conduction and LBBB after ablation might be transient and could be recovered.

RFA of left‐sided accessory pathways can be performed either by a retrograde trans‐aortic approach or transseptal puncture approach. These two methods are possible to result in impairment of AV nodal conduction. Previous case report showed that the development of transient complete AV block was because of the bump injury to the AV node during transseptal catheterization.^1^ It was also possible to result in transient AV block by the retrograde trans‐aortic approach, which produced trauma to the AV node during catheter entry into the LV.^2^

Why did the impairment of AV nodal conduction and transient LBBB occurred during RFA for this patient? It is not clear whether poor AV nodal conduction was intrinsic because it was difficult to measure the AV nodal conduction at baseline EP study under this dominant antegrade accessory pathway. But, since the follow‐up Holter's ECG showed that the HR could reach 125 beats/min, AV nodal 1:1 conduction would be expected to be less than 480 msec. Besides, the baseline ECG showed only minimal preexcited delta wave (Figure 1B). It meant that the baseline AV nodal conduction would be fair during this sinus rhythm (HR = 64/min). This transient impairment of AV nodal conduction and LBBB could be produced by the trans‐aortic approach in which inserted ablation catheter might damage the LBBB and AV node just below the valve or the shaft of the ablation catheter damage the LBBB during manipulation in the LV.^4^ Another possible mechanism was the activation of vagal reflex during the RFA and resulted in conduction impairment of His‐Purkinje system.^5^ But, this vagal reflex would be brief and improve rapidly once the ablation was stopped.

The transient LBBB could also be caused by cardiac memory. Fuenmayor et al^3^ studied 199 patients with WPW who were submitted to RFA. Thirty (15%) exhibited BBB after the ablation. Twenty‐two patients had right BBB (11%) and eight had left BBB (4%). A 46 ± 44 month follow‐up was completed in 24 (80%) of the BBB patients. Twenty three (95.83%) had a normal ECG at the end of the study. The transient nature of the BBB and the lack of association between the site of RFA and the BBB location in this group of WPW syndrome lead us to believe that the BBB is associated with the preexcitation pattern which produced cardiac memory on the bundle branch. Transient BBB might be the result of a cardiac memory phenomenon that induced a longer refractory period of the bundle and a transient BBB that disappears after the cardiac memory reverts.^3^

In conclusion, this rare clinical situation might serve to call attention to the possibility of a transient impairment of AV nodal and His‐Purkinje system during the RFA of the left free‐wall accessory pathway by the trans‐aortic approach.

## CONFLICT OF INTEREST

It is without any conflict of interest.
